# Timing of antibiotic initiation in sepsis and neutropenic fever

**DOI:** 10.3389/fmed.2025.1597047

**Published:** 2025-09-02

**Authors:** Jenny Gretland, Sigrun Sjømæling, Knut Anders Mosevoll, Håkon Reikvam

**Affiliations:** ^1^Faculty of Medicine, University of Bergen, Bergen, Norway; ^2^Department of Medicine, Haukeland University Hospital, Bergen, Norway; ^3^K.G. Jebsen Center for Myeloid Blood Cancer, Department of Clinical Science, University of Bergen, Norway

**Keywords:** sepsis, septic shock, neutropenia, antibiotics, time-to-first-antibiotic, outcome, mortality, *Surviving Sepsis Campaign*

## Abstract

**Introduction:**

Sepsis, as a life-threatening organ dysfunction caused by a dysregulated host response to infection, remains a leading cause of mortality worldwide. The condition requires rapid treatment, and *The Surviving Sepsis Campaign* from 2021 recommend administration of antimicrobials within one hour for suspected septic shock or high likelihood of sepsis.

**Methods:**

We conducted a comprehensive review of the literature regarding timing of antimicrobial administration and its impact on sepsis outcome, to evaluate whether a 1 h target for initiating antibiotics is a reasonable goal. A literature search was conducted in the PubMed database, and we performed a narrative synthesis of the studies.

**Results:**

Of the 42 studies reviewed, 34 demonstrated a significant association between delayed time to first antibiotic and increased mortality rates. The majority of the studies found a significant increase in mortality with delays in antimicrobial initiation, particularly with hourly cutoffs.

**Discussion:**

Sepsis is a heterogeneous condition, complicating the establishment of universal guidelines. Approximately half of the studies identified a near-linear relationship between delayed antimicrobial treatment and increased mortality, emphasizing the benefit of administering antibiotics within 1 h. However, other studies did not observe this linear association, instead reporting significantly increased mortality only after 3–6 h. These findings may indicate that a 1 h goal may not be optimal for all patients.

**Conclusion:**

Based on the findings in this systematic review, the recommendations outlined in *The Surviving Sepsis Campaign* appear to be reasonable goals. For patients with febrile neutropenia, further research is necessary to determine the optimal timing for antimicrobial administration.

## Introduction

“Sepsis is defined as a life-threatening organ dysfunction caused by a dysregulated host response to infection.” Sepsis may progress to septic shock, a condition characterized by circulatory failure and cellular abnormalities, which significantly increases the risk of mortality. Sepsis is a heterogenous condition, depending on age, underlying comorbidities, medications, and source of infection in affected individuals. This heterogeneity makes it challenging to establish standardized treatment guidelines and may account for the differences in outcomes observed across various studies regarding the condition. Sepsis is a leading cause of death from infection, particularly if not recognized and treated promptly, highlighting the urgency of timely diagnosis and intervention (1). Sepsis is a common condition, and its incidence is rising as the global population ages. In a study by Rudd et al. in 2017, it was estimated that nearly 50 million annual cases of sepsis worldwide, and the condition contributes to over 10 million deaths globally, accounting for approximately 1/5 of all deaths worldwide (2).

In 2016, the Third International Consensus for Sepsis and Septic shock (Sepsis-3) updated the definition of sepsis and septic shock (1). The Sepsis 1 and 2 criteria, last reviewed in 2001, focused on systemic inflammation in response to infection, formulating sepsis as a continuum that progresses through sepsis, severe sepsis, and septic shock (3). In contrast, Sepsis 3 incorporated updated knowledge about the pathobiology, epidemiology, and management of sepsis. The definition of Sepsis 3 emphasizes the dysregulated host response to infection, highlighting the clinical heterogeneity among affected individuals and the cellular dysfunction that underlies physiological and biochemical abnormalities in specific organ systems. Additionally, Sepsis 3 considers the term “severe sepsis” redundant, defining sepsis itself as life-threatening organ dysfunction, which may suggest that participants in studies using Sepsis 3 criteria are more severely ill patients (1).

*The Surviving Sepsis Campaign: International Guidelines for Management of Sepsis and Septic Shock* from 2021 provides recommendations for clinicians caring for adult patients with sepsis or septic shock. These guidelines state that antimicrobial therapy is recommended immediately, ideally within one hour of recognition, for adult patients with possible septic shock or a high likelihood of sepsis. For patients with possible sepsis without shock, it is recommended that a rapid, time-limited assessment of the likelihood of infectious versus non-infectious causes and administration of antimicrobials should happen within 3 h of recognition (4).

There is universal agreement with the fact that antimicrobial therapy should be delivered as early as possible in sepsis treatment. However, the exact timepoint for delivery remains controversial. In this article, we review the literature regarding the timing of antimicrobial administration and its impact on septic patients to evaluate whether a one-hour target for starting antibiotics is reasonable.

## Materials and methods

The systematic review is reported following the Preferred Reporting Items for Systematic Reviews and Meta-Analyses (PRISMA) checklist from 2020 (5).

### Eligibility criteria

#### Types of studies and population

We included published randomized clinical trials (RCTs), retrospective cohort studies (RCS), and prospective cohort studies (PCS) that involved adult patients diagnosed with sepsis, bloodstream infections, or neutropenic fever. Studies were excluded if they did not mention sepsis or neutropenic fever in their title or abstract.

#### Intervention

The intervention of interest was antibiotic treatment, explicitly focusing on the time to first antibiotic administration (TTFA). Studies were excluded if the primary intervention was not antibiotic administration, such as those evaluating vasopressor treatment, surgical interventions in septic patients and evaluations of sepsis screening tools.

#### Outcome

We included studies that reported on patient outcomes related to the time to administration of the first dose of antibiotics. The primary outcomes of interest were mortality rates and progression to septic shock.

Studies were divided into two groups for synthesis: one focusing on the TTFA in adult septic patients and one focusing on the TTFA in septic neutropenic patients, with both groups investigating patient outcomes.

### Information sources and search strategy

We conducted a literature search using the PubMed database. Collaborating with a biomedical librarian, we formulated a search strategy based on relevant keywords and medical terms aligned with our eligibility criteria. Initially, we performed a simple search using “sepsis hours antibiotics” to estimate the volume of literature, yielding 2919 results. We then refined our search terms using Medical Subject Headings (MeSH). The initial MeSH terms included “sepsis” OR “bacteremia” OR “Shock, Septic” AND “Anti-Bacterial Agents” AND “Time-to-treatment.” By applying these MeSH terms in PubMed, we identified additional relevant terms. The comprehensive search strategy is detailed in Supplementary Table 1.

The original search was conducted on 11 October 2023, and yielded 235 results. After one duplicate was removed, 234 studies remained. To ensure the search was up to date prior to publication, we updated the literature search on 19 May 2025, and got 350 results in total. Additionally, we examined the reference lists of the excluded systematic reviews and consulted the Surviving Sepsis Campaign guidelines to identify further studies that met our inclusion criteria (4, 6–9).

### Selection process

The remaining records were imported into Rayyan for a double-blinded selection process based on our predefined inclusion and exclusion criteria (10). After unblinding the selections, the authors reviewed the articles and identified 26 studies that met the inclusion criteria. Any disagreements between the authors were resolved through discussion. The excluded articles were categorized according to the most appropriate exclusion criteria, as detailed in Supplementary Figure 1.

As the Surviving Sepsis Campaign guidelines form the foundation for sepsis treatment, we conducted a double-blinded selection of its references. Furthermore, we excluded five systematic reviews from the literature search and examined their reference lists for relevant studies. Articles were included upon mutual agreement. This process added 16 more studies to our literature list. A detailed flow chart, inspired by the PRISMA 2020 flow chart, illustrating the study selection process is provided in Supplementary Table 2 (5).

### Data collection process and data items

The two reviewers developed a comprehensive data extraction form to collect study characteristics from the included studies systematically. This included authors, study design, year of publication, sample size studied, sepsis definition used, patient outcome studied, and whether the outcomes were significantly associated with TTFA. Additionally, we recorded the median time to antibiotics and specified the timepoints for antibiotic administration investigated.

Reviewers independently extracted all information aligned with the predefined study characteristics to ensure accuracy and reduce bias. Discrepancies between reviewers were resolved through discussion or by consulting our supervisors. For studies with missing information, such as median time to antibiotics, we noted them as “not demonstrated.”

In cases where studies did not provide a clear sepsis definition, we reviewed the inclusion criteria provided by the authors and classified the studies based on whether their criteria resembled with the Sepsis 1 and 2 definitions or the Sepsis 3 definition. These studies were included under the Sepsis 1 and 2 definitions: Whiles et al. (11), Bloos et al. (12), Ferrer et al. (13), Pruinelli et al. (14) were all investigating patients with severe sepsis, a terminology used in the Sepsis 1 and 2 definitions. Rhee et al. (15) defined sepsis by infection plus two SIRS criteria (-s) or organ dysfunction, in line with the former definition. Sivayoham et al. (16) included patients who met either two SIRS-criteria or at least one Red Flag Sepsis criteria from the United Kingdom Sepsis Trust on arrival, aligning with the Sepsis 1 and 2 criteria. Two studies with initially uncertain definition were classified under the Sepsis 3: Rüddel et al. (17), Tantarattanapong et al. (18). Rüddel et al. (17) defined sepsis as suspected infection with organ dysfunction, without requiring the presence SIRS criteria. Tantarattanapong et al. (18) defined sepsis as suspected infection combined with a National Early Warning Score ≥ 5 points, but enrolled patients through a sepsis protocol based on the 2016 Surviving Sepsis Campaign guidelines, which use the Sepsis 3 definition.

### Study risk of bias assessment

Risk of bias in the included studies was assessed using the Newcastle-Ottawa Scale (19). The scale evaluates studies based on three broad perspectives: selection of study groups, comparability of the groups, and ascertainment of the outcome of interest for cohort studies, with a maximum score of nine stars. Studies scoring 7–9 stars were categorized as high quality, 4–6 stars as moderate quality, and 0–3 stars as low quality, based on prior usage in similar systematic reviews (7). The two authors independently assessed the risk of bias for each included study. After completing the separate assessments, we compared our results and discussed any discrepancies to reach a consensus.

### Effect measures

For each outcome assessed in this systematic review, we used the following effect measures to present the results: odds ratios (OR), hazard ratios (HR) and percentages, along with their 95% confidence interval (CI) and *p*-values when available.

The specific outcomes included:

- Different forms of mortality, such as in-hospital mortality, 28 days mortality, 30 days mortality, 90 days mortality and 1 year mortality
- Progression to septic shock

These measures were selected based on the nature of the data available from the included studies.

### Synthesis methods

For the synthesis, studies were categorized into two groups based on the sepsis definition used: one for Sepsis 1 + 2 definition and another for Sepsis 3 definition. Additionally, a separate group was created for studies focusing on neutropenic sepsis patients. Due to high heterogeneity between patients in the different studies, these groups were defined to ensure that studies with similar diagnostic criteria and patient populations were analyzed together, providing a more homogeneous basis for synthesis. This approach also accounts the differences in disease severity among participants, as studies using different definitions may reflect varying clinical profiles.

When preparing the data for synthesis, only a few data required adjustments. For studies that reported time points in minutes, data were converted into hours to ensure consistency across included studies. For studies that reported different outcome measures, we maintained their original results as presented by the authors and did no adjustments.

We organized the studies into nine structured tables: eight based on the Sepsis 1 + 2 and Sepsis 3 definitions, and one for studies focusing on neutropenic sepsis. The sepsis definitions represent a difference in disease severity in patients with sepsis, and we aimed to investigate whether there were any variations in results based on these definitions. The table for patients with neutropenic sepsis aimed to investigate whether patients with immunodeficiency, such as those undergoing chemotherapy, showed a trend toward an increased risk of adverse outcomes due to delays in administering antibiotics.

The tables were organized according to timepoints and sample sizes, and included the studied patient outcome, whether there was a significant association between TTFA and outcomes (marked in green, yellow, or red). To assess the accuracy of the results, we also noted whether the studies differentiated between sepsis, severe sepsis and septic shock, as well as the median time to antibiotics. For each study, we created a separate column with detailed statistical information, such as odds ratios.

Due to the high heterogeneity among studies and outcome measures, we chose not to conduct a meta-analysis. Instead, we used a narrative synthesis to describe the findings qualitatively and summarize the patterns observed in each study. This method provided a flexible approach to comparing results across studies, making it easier to identify patterns between TTFA and outcomes despite the high heterogeneity. We then compared these findings with the recommendations from the Surviving Sepsis Campaign.

When synthesizing our results, we compared studies investigating similar specific time points. For example, some studies focused on the impact of each hourly delay in TTFA on patient outcomes, while others examined delays before and after a 3 h threshold. We evaluated whether most of these studies demonstrated a statistically significant difference in outcomes or not. By comparing specific time points and outcomes we reduced potential sources of variability in our results. For studies with both significant and non-significant results, we provided explanations in the table or labeled them as “Yes, regarding certain time points.” Non-significant results were marked as “NS.”

Furthermore, when comparing our findings to the Surviving Sepsis Campaign guidelines, we assessed whether our results indicated a trend of increased mortality with each additional hour of delay in septic shock patients, as well as a trend related to the three-hour threshold for septic patients overall (4).

### Reporting bias and certainty assessment

We did not assess reporting bias and certainty due to the nature of our synthesis. Nevertheless, we acknowledge that reporting bias, variability in outcome measures and sample sizes could have influenced our results.

## Results

### Study characteristics

Tables 1–3 present the studies’ characteristics, effect measures and outcomes. The studies are, as stated, divided by sepsis definition, as well as timepoints studied. Tables 1a–d present studies using the sepsis 1- and 2-definitions and are sorted by time to antimicrobial initiation: hourly cutoffs, < 1 h <, < 3 h < and lastly < 6 h < and other timepoints studied, respectively. Tables 2a–d present studies using the sepsis 3-definition, whereas Table 3 presents the studies of septic neutropenia. Both retrospective and prospective cohort studies, and one cluster randomized trial were included, from 2006 to 2025. The sample size varied from 90 to 74,114 adult participants with sepsis, severe sepsis, septic shock, and septic neutropenia.

**TABLE 1a:** Presents the characteristics of studies using the Sepsis 1 and 2 definitions, assessing hourly time to first antibiotic (TTFA) and outcomes.

References	Sample size	Patient outcome	Does the study differentiate between sepsis, severe sepsis, and septic shock?	Significant association between TTFA and outcome?	Median time to AB (h)	Details, OR
Bisarya et al. RCS/2022 (34)	74,114	In-hospital mortality Progression to SSh	No	Yes	1.85	In-hospital mortality: aOR 1.02 (95% CI, 1.006–1.04; *P* = 0.007) Progression to SSh: aOR 1.04 (95% CI, 1.03–1.04; *P* < 0.001)
Yang et al. RCS/2023 (35)	55,169	In-hospital mortality	No	Yes	0.9 (0.3–1.8)	aOR = 1.03 (95% CI, 1.02–1.04; *P* < 0.001)
Seymour et al. RCS/2017 (36)	49,331	In-hospital mortality	Yes, studies severe sepsis and SSh	Yes	0.95 (0.35–1.95)	OR = 1.04 (95% CI 1.03–1.06; *P* < 0.001)
Liu et al. RCS/2017 (37)	35,000	In-hospital mortality	Yes	Yes	2.1 (1.4–3.1)	All sepsis patients: aOR 1.09 (95% CI, 1.05–1.13; *P* < 0.001) Sepsis: aOR 1.09 (95% CI, 1.00–1.19; *P* = 0.046) Severe sepsis: aOR 1.07 (95% CI, 1.01–1.24; *P* = 0.014) SSh: aOR 1.14 (95% CI, 1.06–1.23; *P* = 0.001)
Bloos et al. RCT/2017 (12)*	4,183	28 days mortality	Yes, studies severe sepsis and SSh	Yes	Control group: 2.0 (0.4–5.9) Intervention group: 1.5 (0.1–4.9)	OR = 1.02 (95% CI, 1.01–1.03; *P* < 0.001)
Whiles et al. RCS/2017 (11)*	3,929	Progression to SSh In-hospital mortality	Yes, studies sepsis and SSh	Yes	3.13 (1.79–5.53)	In-hospital mortality: OR = 1.05 (95% CI, 1.03–1.07) Progression to SSh: OR = 1.08 (95% CI, 1.06–1.10; *P* < 0.001)
Kumar et al. RCS/2006 (26)	2,731	In-hospital mortality	Yes, studies SSh and TTFA after onset of hypotension	Yes	6 (2.0–15.0)	aOR 1.119 (95% CI 1.103–1.136; *P* < 0.0001)
Abe et al. PCS/2019 (27)	1,124	In-hospital mortality	Yes, studies severe sepsis and SSh	No	1.7 (0.9–3.15)	OR 0.999 (0.997–1.000; *P* = 0.152)
Corl et al. RCS/2019 (38)	506	30 day mortality	Yes, studies SSh	Yes	3 (1–5)	OR 1.11 (95% CI, 1.02–1.21; *P* = 0.01)
Peltan et al. RCS/2017 (46)	421	In-hospital mortality	No	Yes	1.97 h (1.37–3.0)	OR: 1.20, (95% CI 1.00–1.44; *P* = 0.046)
Suberviola et al. PCS/2015 (39)	342	In-hospital mortality	Yes, studies SSh	Yes	1.7 (0–5.5)	OR 1.03 (95% CI 1.01–1.06; *P* = 0.008)
Puskarich et al. PCS/2011 (20)	291	In hospital mortality	Yes, studies SSh	No	1.92 (1.1–2.92)	No hourly significant association between mortality and TTFA within the first 6 h. TTFA before and after shock recognition: aOR 2.49 (95% CI 1.7–5.74)
Garnacho-Montero et al. PCS/2006 (40)	224	In-hospital mortality	Yes, studies sepsis and SSh	Yes	ND	Sepsis: OR 1.09 (95% CI 1.04–1.14) SSh: OR 1.06 (95% CI 1.01–1.10)

*Unspecified sepsis definition. ND, not demonstrated, SSh, septic shock.

**TABLE 1b:** Presents studies using the Sepsis 1/2 definitions, comparing outcomes between time to first antibiotic (TTFA) within 1 h versus beyond 1 h.

Reerences	Sample size	Patient outcome	Does the study differentiate between sepsis, severe sepsis, and septic shock?	Significant association between TTFA and outcome?	Median time to AB (h)	Details, OR
Ko et al. PCS/2020 (47)	2,250	In-hospital mortality	Yes, studies SSh	Yes	2.29 (1.42–3.5)	OR 1.367 (95% CI 1.177–1.589; *P* < 0.001)
Sivayoham et al. RCS/2020 (16)*	2,066	In-hospital mortality	No	Yes, but only in patients with RH	0.8 (0.5–1.36)	Overall population: aOR 1.00 (0.99–1.00; *P* = 0.89) Patients with RH and AB administered later than 55 min after arrival in ED: OR 2.75 (95% CI, 1.22–6.14)
Bulle et al. RCS/2021 (31)	1,587	90 days mortality	Yes, studies SSh	No	1.15 (0.53–1.87)	aOR 1.30 (95% CI 0.95–1.76; *P* = 0.10)
Londoño et al. PCS/2018 (48)	884	In-hospital mortality	No	Yes	4.0 (2–7)	Reduction in mortality of 30% (*P* = 0.024)
Gaieski et al. RCS/2010 (21)	261	In hospital mortality	Yes, studies severe sepsis and SSh	Yes	1.98 (1.27–3.2)	Time from ED triage to appropriate AB administration, < 1 h <: OR 0.30 (95% CI 0.11–0.83; *P* = 0,02)

*Unspecified sepsis definition. AB, antibiotic therapy; ED, emergency department; RH, refractory hypotension; SSh, septic shock.

**TABLE 1c:** Presents studies using the Sepsis 1/2 definitions, comparing outcomes between time to first antibiotic (TTFA) within 3 h versus beyond 3 h.

References	Sample size	Patient outcome	Does the study differentiate between sepsis, severe sepsis, and septic shock?	Significant association between TTFA and outcome?	Median time to AB (h)	Details, OR
Seymour et al. RCS/2017 (36)	49,331	In-hospital mortality	Yes, studies severe sepsis and SSh	Yes	0.95 (0.35–1.95)	< 3 h vs 3–12 h: OR 1.14 (95% CI 1.06–1.22; *P* = 0.001)
Pruinelli et al. RCS/2018 (14)*	5,072	In-hospital mortality	Yes, studies severe sepsis or SSh	Yes	ND	Graphic display of effect measure.
Ko et al. PCS/2020 (47)	2 250	In-hospital mortality	Yes, studies SSh	Yes	2.29 (1.42-3.5)	> 3 h <: OR 1.324 (95% CI 1.148–1.529; *P* < 0.001)
Londoño et al PCS/2018 (48)	884	In-hospital mortality	No	Yes	4.0 (2–7)	Reduction in mortality of 21% (*P* = 0.045)
Rhee et al. RCS/2019 (15)*	851	In-hospital mortality	Yes, studies severe sepsis and SSh	Yes	ND	aOR 1.94 (95% CI 1.04–3.62, *P* = 0.038)
Joo et al. RCS/2014 (49)	591	In hospital mortality	Yes, studies severe sepsis and SSh	Yes	1.9 (1.4–2.4)	aOR 0.54 (95% CI 0.34–0.87; *P* = 0.01)
Gaieski et al. RCS/2010 (21)	261	In hospital mortality	Yes, studies severe sepsis and SSh	No	1.98 (1.27–3.2)	Time from ED triage and TTFA: No significant association.

*Unspecified sepsis definition. ED, emergency department; ND, not demonstrated; SSh, septic shock.

**TABLE 1d:** Presents studies using the Sepsis 1/2 definitions, comparing outcomes between time to first antibiotic (TTFA) within 6 h versus beyond 6 h and other time points studied.

References	Sample size	Patient outcome	Does the study differentiate between sepsis, severe sepsis, and septic shock?	Significant association between TTFA and outcome?	Median time to AB (h)	Details, OR
Ferrer et al. PCS/2009 (13)*	2,796	In-hospital mortality	Yes, studies severe sepsis and SSh	Yes	ND	Broad-spectrum AB < 1 h vs. no AB < 6 h: aOR 0.67 (95% CI, 0.50–0.90; *P* = 0.008)
Schinkel et al. RCS/2021 (53)	2,672	28 days mortality	No	Yes	1.6 (0.6–2.1)	Antibiotics received in a prehospital setting: OR = 0.07 (95% CI, 0.01–0.79; *P* = 0.031)
Puskarich et al. PCS/2011 (20)	291	In hospital mortality	Yes, studies SSh	Yes, before and after shock recognition	1.92 (1.1–2.92)	No hourly significant association between mortality and TTFA within the first 6 h. TTFA before and after shock recognition: aOR 2.49 (95% CI 1.7–5.74)
Gaieski et al. RCS/2010 (21)	261	In hospital mortality	Yes, studies severe sepsis and SSh	No	1.98 (1.27–3.2)	Time from ED triage and TTFA at different hourly cutoffs: No significant association, except < 1 h <.
Wisdom et al. RCS/2015 (30)	220	In-hospital mortality	Yes, studies sepsis and severe sepsis	No	3.5 (1.7–6.6)	Uncomplicated sepsis: No association between increased delay to antibiotics and mortality. Severe sepsis: Increased mortality with delays in TTFA with more than 6 h compared with TTFA within 1 h: HR 2.25 (95% CI 0.91–5.59; *P* = 0.08)
Andersson et al. RCS/2019 (52)	90	28 days mortality	Yes, studies severe sepsis and SSh	Yes	ND	Studied the effect of 1^st^ dose antibiotic treatment given < 1 h, and 2^nd^ dose with less than 25% delay. OR 0.096 (95% CI 0.011–0.846; *P* = 0.035)

*Unspecified sepsis definition. AB, antibiotic therapy; ED, emergency department; ND, not demonstrated; SSh, septic shock.

**TABLE 2a:** Presents studies using the Sepsis 3 definitions, assessing hourly time to first antibiotic (TTFA) and outcomes and other time points studied.

References	Sample size	Patient outcome	Does the study differentiate between sepsis, severe sepsis, and septic shock?	Significant association between TTFA and outcome?	Median time to AB (h)	Details, OR
Han et al. RCS/2021 (41)	60,817	In-hospital mortality	No	Yes	3.4 (2.0–6.3)	Order delay: aOR 1.04 (95% CI, 1.03–1.05; *P* < 0.001) Delivery delay: aOR 1.05 (95% CI, 1.02–1.08; *P* < 0.001) Cumulative delay: aOR 1.04 (95% CI, 1.03–1.05; *P* < 0.001)
Peltan et al. RCS/2019 (22)	10,811	1 year mortality	No	Yes	2.8 (1.9–3.8)	Every h: aOR 1.10 (95% CI, 1.05–1.14; *P* < 0.001)
Rüddel et al. RCS/2022 (17)*	4,792	28 days mortality Progression severe sepsis to septic shock	Yes, studies sepsis and SSh	Yes	2.5 (1.0–6.3)	28 days mortality: Overall population: OR 1.019 (95% CI 1.01–1.028; *P* ≤ 0.001) Sepsis: OR 1.026 (95% CI 1.01–1.043) SSh: OR 1.018 (95% CI 1.008–1.029) Progression from SS to SSh: OR 1.051 (95% CI 1.022–1.081; *P* < 0.001)
Im et al. RCS/2022 (23)	3,035	In-hospital mortality	Yes, studies sepsis and SSh	Yes	2.35 (1.3–3.8)	Every h: OR 1.35 (95% CI, 1.01–1.81; *P* = 0.042)
Seymour et al. RCS/2017 (42)	2,683	In-hospital mortality	No	Yes	3.6 (2.1–7.5)	Total medical contact delay (time from first healthcare contact to antibiotic administration): OR 1.03 (95% CI, 1.00–1.05; *P* < 0.01) Emergency department delay (time from arrival ED to antibiotic administration): OR 1.03 (95% CI, 1.00–1.05, *p* = 0.04)
Philippon et al. RCS/2024 (45)	791	28 days mortality	No	Yes	1.02 (0.23–2.82)	Total population: OR 1.06 (95% CI 1.02–1.10) Sepsis without hypotension: aOR 2.02 (95% CI, 1.08–3.76)
Tantarattanapong et al. /RCS/2021 (18)*	600	In-hospital mortality	No	No	0.85 (0.6–1.48)	*P* = 0.866

*Unspecified sepsis definition. SS, severe sepsis; SSh, septic shock.

**TABLE 2b:** Presents studies using the Sepsis 3 definition, comparing outcomes between time to first antibiotic administration (TTFA) within 1 h versus beyond 1 h.

References	Sample size	Patient outcome	Does the study differentiate between sepsis, severe sepsis, and septic shock?	Significant association between TTFA and outcome?	Median time to AB (h)	Details, OR
Peltan et al. RCS/2019 (22)	10,811	1 year mortality	No	No	2.8 (1.9–3.8)	aOR 1.26 (95% CI 0.98–1.62; *P* = 0.07)
Im et al. RCS/2022 (23)	3,035	In-hospital mortality	Yes, studies sepsis and SSh	Yes, but not in patients without shock	2.35 (1.3–3.8)	Overall population: aOR 0.78 (95% CI, 0.61–0.99; *P* = 0.046) Sepsis without shock: aOR 0.85 (95% CI, 0.64–1.15; *P* = 0.300) Sepsis with shock: aOR = 0.66 (95% CI, 0.44–0.99; *P* = 0.049)
Philippon et al. RCS/2024 (45)	791	28 days mortality	No	Yes	1.02 (0.23–2.82)	aOR 2.00 (95% CI, 1.24–3.23)
Tantarattanapong et al. /RS/2021 (18)*	600	In-hospital mortality	No	No	0.85 (0.6–1.48)	≤ 1 h and > 1 h (13.1% vs. 11.6%; *P* = 0.726).
Seok et al. PCS/2020 (29)	482	7-, 14- and 28 days mortality	No	No	1.9 (1.27–3.45)	The time to initial antibiotics were not associated with 7-, 14- and 28 days mortality in multivariate analysis.
Liang et al. /RS/2022 (32)	331	90 days mortality	Yes, studies SSh	No	1.35 (0.58–2.0)	≤ 1 h and > 1 h (44.2% vs. 43.5%; *P* = 0.90)

*Unspecified sepsis definition. SSh, septic shock,

**TABLE 2c:** Presents studies using the Sepsis 3 definition, comparing outcomes between time to first antibiotic administration (TTFA) within 3 h versus beyond 3 h.

References	Sample size	Patient outcome	Does the study differentiate between sepsis, severe sepsis, and septic shock?	Significant association between TTFA and outcome?	Median time to AB (h)	Details, OR
Peltan et al. RCS/2019 (22)	10.811	1 year mortality	No	Yes	2.8 (1.9–3.8)	< 3 h <aOR 1.27 (95% CI, 1.13–1.43; *P* < 0.001)
Philippon et al. RCS/2024 (45)	791	28 days mortality	No	Yes	1.02 (0.23–2.82)	aOR 2.48 (1.40–4.40)
Seok et al. PCS/2020 (29)	482	7-, 14- and 28 days mortality	No	No	1.9 (1.27–3.45)	The time to initial antibiotics were not associated with 7-, 14- and 28 days mortality in multivariate analysis.
Ogawa et al. RCS/2023 (24)	93	In-hospital mortality 30 days mortality	Yes, studies sepsis and SSh in patients with perforated colorectal peritonitis	No	2.3	Logistic regression analysis was used to extract ORs for cut-off values of TTFA at 120, 162, and 180 min. Only a TTFA ≥ 162 min was predictive of the hospital-stay mortality, 30 days mortality, and 60 days mortality.

SSh, septic shock.

**TABLE 2d:** Presents studies using the Sepsis 3 definition, comparing outcomes between time to first antibiotic administration (TTFA) within 6 h versus beyond 6 h and other timepoints studied.

References	Sample size	Patient outcome	Does the study differentiate between sepsis, severe sepsis, and septic shock?	Significant association between TTFA and outcome?	Median time to AB (h)	Details, OR
Taylor et al. RCS/2021 (51)	28,865	In-hospital mortality	No	Yes	3.4 (2.0–6.0)	Triage to order time > 6 h vs. < 1 h: OR 1.21 (95% CI, 1.0–1.46; *P* < 0.01) Order to infusion time of 1.5–2 h compared to < 0.5 h: OR 1.35 (95% CI, 1.07–1.71) Order to infusion time of 2–2.5 h compared to < 0.5 h: OR 1.51 (95% CI, 1.14–2.00; *P* < 0.01) Both recognition delay and administration delay were associated with mortality.
Rüddel et al. RCS/2022 (17)*	4,792	28 days mortality Progression severe sepsis to SSh	Yes, studies sepsis and SSh	Yes, regarding certain time points	2.5 (1.0–6.3)	28 days mortality: OR 1.41 (95% CI 1.17–1.69) No significant differences comparing treatment within 1 h versus 1–3 h, or 1 h versus 3–6 h.
Siewers et al. PCS/2021 (50)	590	28 days mortality	No	Yes	4.7 (2.7–8.1)	TTFA was categorized into different time-intervals. Lowest mortality observed in patients with TTFA 3–6 h, therefore used as reference. Lowest observed mortality in patients who received AB between 1–9 h. ≤ 1 h = OR 3.06 (1.25–7.46) 1–3 h = aOR 1.67 (0.83–3.37) 6–9 h = aOR 1.17 (0.56–2.49) > 9 h = aOR 1.91 (0.96–3.85)
Seok et al. PCS/2020 (29)	482	7-, 14- and 28 days mortality	No	No	1.9 (1.27–3.45)	The time to initial antibiotics were not associated with 7-, 14- and 28 days mortality in multivariate analysis.
Ogawa et al. RCS/2023 (24)	93	In-hospital mortality 30 days mortality	Yes, studies sepsis and SSh in patients with perforated colorectal peritonitis	Yes, regarding certain time points	2.3	Logistic regression analysis was used to extract ORs for cut-off values of TTFA at 120, 162, and 180 min. Only a TTFA ≥ 162 min was predictive of the hospital-stay mortality, 30 days mortality, and 60 days mortality. In-hospital mortality: < 162 min < OR 7.1 (95% CI 1.64–49; *P* = 0.007) 30 days mortality: All sepsis patients: < 162 min < OR 10 (95% CI 1.6–195; *P* = 0.01) Septic shock: < 162 min < OR 12 (95% CI, 1.47–257; *P* = 0.019)

*Unspecified sepsis definition. AB, antibiotic therapy; SSh, septic shock.

**TABLE 3:** Presenting the characteristics and outcomes of the studies focusing on neutropenic sepsis.

References	Sample size	Patient outcome	Does the study differentiate between sepsis, severe sepsis, and septic shock?	Significant association between TTFA and outcome?	Median time to AB (h)	Timepoint for antibiotics studied					Details, OR/RR/HR
						Every h	< 1 h	<3 h	< 6 h	Other	
Daniels et al. RCS/2019 (25)	3.219	30 days mortality	No	Yes, regarding certain time points	ND					X	When compared to patients receiving AB within 2 h of fever as reference: 3–6 h: OR 1.57 (*P* = 0.04) 6–24 h: 1.37 (*P* = 0.11), non-significant 24–48 h: OR 2.08 (*P* = 0.02)
Ko et al. RCS/2015 (28)	1.001 admissions with 863 patients	In hospital mortality	Yes, studies severe sepsis and SSh	No	2.3 (1.83–3.0)		NS	NS		NS	Failed to find a significant relationship between AB timing and mortality in febrile neutropenia and severe sepsis/septic shock in patients receiving AB in ≤ 1 h vs. > 1 h, ≤ 2 h vs. > 2 h, ≤ 3 h vs. > 3 h, and ≤ 4 h vs. > 4 h.
Goldman et al. RCS/2017 (43)	162	Progression to SSh	Yes, studies severe sepsis and septic shock	Yes	1.85 (1.0–1.97)	X					aOR 1.18 (95% CI 1.01–1.38; *P* = 0.03)
Isaranuwatchai et al. RCS/2025 (33)	149	28 days mortality	No, studies patients with confirmed diagnosis of febrile neutropenia	No	0.83 (0.58–1.12)		NS				≤ 1 h and > 1 h (1.71% vs. 6.25%; *P* = 0.202)
Morneau et al. RCS/2017 (44)	100	In-hospital mortality	No	Yes	For survivors: 3.4 (2.1–11.1) For non-survivors: 9.8 (0.5–118.7)	X					Overall population: aOR 1.16 (95% CI 1.04–1.34; *P* = 0.04) Culture-positive patients: aOR 1.25 (95% CI 1.03–1.53; *P* = 0.04)

ND, not demonstrated; NS, not significant; SSh, septic shock.

Different studies investigated different outcomes, various timepoints for antibiotic administration, and the risk of outcomes arising. Some provided an odds ratio for every hour of delay, some compared administration before and after 1, 3, and 6 h, and others studied different timepoints. Outcomes varied between in-hospital mortality, 7-, 14-, 28- or 30 days mortality, 1 year mortality and risk of progression to septic shock. When the study investigated several different outcomes, the primary outcome was prioritized in each study.

### Time to antibiotics and outcome

Of the total of 42 articles included in this study, eight studies found a partial association between TTFA and mortality (16, 17, 20–25). This indicates that an association was proved only regarding specific time points or patient subgroups. Peltan et al. (22) observed that each one-hour increase in door-to-antibiotic time was associated with a 10% increase in the odds of 1 year mortality, and similarly for in-hospital, 30 and 90 days mortality. A significant association between TTFA and mortality was also seen when antimicrobial agents were received before versus after 3 h (aOR 1.27 (95% CI, 1.13–1.43; *P* < 0.001), but the same association was not seen regarding a 1 h time point (22). Gaieski et al. (21) found a significant increase in mortality when antimicrobials were administered before and after 1 h, but no significant association was found when assessing hourly cutoffs. Puskarich et al. (20) discovered a significantly increased mortality in patients who received antimicrobials after shock recognition, but mortality did not change with hourly delays in antibiotic delivery. A study on patients with febrile neutropenia by Daniels et al. (25) found that while mortality was lower in those receiving antimicrobials within 2 h compared to the 3–6 and 24–48 h periods, this association was not observed in patients receiving antimicrobials between 6 and 24 h. Kumar et al. (26), Sivahoyam et al. (16) proved a significant association between TTFA and mortality in patients with recurrent or persistent hypotension, and Im et al. (23) in patients with septic shock. Rüddel et al. (17), illustrate a significant increase in mortality for every hour of delay in TTFA but reports non-significant differences when comparing treatment within 1 h to treatment within 1–3 or 3–6 h.

Eight studies failed to overall find a statistically significant association between TTFA and mortality (18, 27–33). The findings in a study conducted by Wisdom et al. (30) suggest an increased risk of mortality with increased delay in antibiotic timing in patients with severe sepsis. A hazard ratio exceeding two was observed when antibiotic administration exceeded 6 h compared to 1 h, and though not statistically significant, a doubling in risk of mortality is clinically meaningful (30). This trend was not seen among patients with uncomplicated sepsis (30).

Different time points were used for the studies that proved a significant correlation between TTFA and mortality. Nineteen studied the mortality with hourly delay in antimicrobial administration (11, 12, 17, 22, 23, 26, 34–46). Six studies found increased mortality with a 1 h delay in antimicrobial delivery (16, 21, 23, 45, 47, 48). Eight found an association between mortality and time to antibiotics administered after a 3 h threshold (14, 15, 22, 36, 45, 47–49). Siewers et al. (50) found the lowest mortality in the group who received antimicrobials between 3 and 6 h. Wisdom et al. (30), Taylor et al. (51) assessed the mortality of patients receiving antimicrobials after 6 h compared to before. Some studies have also used other time points, which are specified in the columns for effect measures.

### Risk of bias assessment

For evaluating the risk of bias in studies included, we used the Newcastle-Ottawa scale (19), and details of the quality assessment are in Supplementary File 3. With a maximum score of nine, each study scored six or higher, indicating that the studies included are of moderate to high quality. The average score was above eight.

## Discussion

### Main findings

Based on the findings of the included studies, the need for timely antibiotic therapy is obvious when treating septic patients. 34 out of 42 of the studies included found a significant association between TTFA and mortality, proving its importance (11–17, 20–26, 34–53). Nevertheless, the last eight studies failed to do so (18, 27–33). Wisdom et al. (30) found a clinically significant association between mortality and TTFA amongst patients with complicated sepsis, although not statistically significant. Though the need for timely antibiotic treatment is evident, this study seeks to get an overview of studies presenting data for when antimicrobial therapy should be initiated. The heterogeneity of sepsis manifestation could explain why the studies have such varying results. This heterogeneity, along with various time points studied, provides a challenge when deciding a cutoff time for when antimicrobials should be received as part of sepsis treatment. There are several things we have put into consideration.

First, we assessed the studies proving the benefit of early antimicrobials and those who failed to do so. The majority of the studies that demonstrated a significant association between TTFA and mortality, as well as those that did not, were of high quality in regard to the Newcastle-Ottawa scale (19). Only two studies were of moderate quality (18, 19, 45). The studies with the most significant number of participants, close to 30,000 or more, all found a significant association between TTFA and mortality (34–37, 41, 51). The studies with the most significant number of participants, close to 30,000 or more, all found a significant association between TTFA and mortality (34–37, 41, 51). More extensive studies include more vast patient characteristics, thus providing a more representative patient group, leading to a potentially more reliable study.

Six studies failed to find a significant association between TTFA and mortality (18, 27, 29–32). These are all relatively small studies with 331–1,587 participants. Abe et al. (27), Seok et al. (29), Liang et al. (32), Tantarattanapong et al. (18), had a short median TTA, with 102, 115, 81 and 51 min, respectively, indicating that its participants received antimicrobials at an early stage, leaving smaller grounds for comparison when the low number of participants is also considered. Liang et al. (32), Tantarattanapong et al. (18) studied exclusively elderly patients with high comorbidity and mortality risk. They compare TTFA > 1 h to ≤ 1 h, but all received antibiotics within 2 h, limiting the time range and comparability (18, 32). The same applies to Bulle et al. (31), where all patients received antibiotics within 112 min. In the study of Abe et al. (27), patients who received antimicrobials within 1 h had the highest mortality rate. A possible explanation is that these patients had the most severe clinical presentations and therefore received earlier recognition and treatment. However, the severity of illness may have led to death regardless of TTFA. Abe et al. (27) also note that an earlier study report that TTFA was related to better outcomes if patients receiving antimicrobials within one hour were excluded.

Second, we considered the different delay-time points proven to have a significant association with mortality. Just below half of the articles, including almost all the largest studies, found a near-linear model with increasing mortality due to every hour delay in initiating of antimicrobial treatment, indicating that every hour without treatment counts (11, 12, 17, 22, 23, 26, 34–42, 45, 46). The majority of articles studying delay with hourly cutoffs specifically, found a significant increase in mortality. However, crucial to keep in mind that the hourly increment in mortality because of delayed antimicrobial therapy results from linear models, which may not reflect an actual stepwise increase in mortality per hour. In addition, six out of eleven articles studying mortality with TTFA being delayed by 1 h found a significant association (16, 21, 23, 45, 47, 48). Schinkel et al. (53) found reduced mortality when antibiotic therapy was initiated in a pre-hospital setting. When assessing outcomes in studies conducted both with hourly delays and with a 1 h-cutoff, it is evident that antimicrobial therapy initiated before a 1 h mark effectively improves septic patients’ outcomes. We also considered the studies focusing on the remaining time points. Eight out of eleven studies found significantly increased mortality first when antimicrobials were received after 3 h (14, 15, 22, 36, 47, 49). Ogawa et al. (24) found a significant association between mortality and TTFA being later than 162 min, close to 3 h. Siewers et al. (50) found the lowest mortality when antimicrobials were received between 3 and 6 h. Taylor et al. (51) found a similar association regarding an order delay of 6 h. These findings may indicate that a 1 h goal may not always be optimal. However, they support the 3 h limit when a sepsis diagnosis is uncertain. It is important to keep in mind that this review is based on studies that have explicitly reported results of time points to antimicrobial initiation. We acknowledge that studies reporting only positive findings might be missed.

Third, we aimed to investigate a potential association regarding severity of disease and TTFA, to see if this had impact on mortality. This proved challenging due to the fundamental differences in patient groups included in each of the different sepsis definitions.

As for the studies regarding septic neutropenia, only five were included, and three out of five found a significant association between TTFA and mortality (25, 28, 33, 43, 44). Daniels et al. (25) found a significant association only regarding specific time points. Sung et al. (28), with 1,001 admissions and 863 patients, contribute with the second biggest of the studies regarding neutropenic sepsis, but they failed to prove a significant association. In the study of Daniels et al. (25) 45.1% of the patients had a clinical diagnosis of infection, and only 22% had a bloodstream infection. The same trend is seen in the other included studies, where only a percentage of the patients present a clinical sepsis diagnosis (43). Mortality was rare in the study of Goldman et al. (43). Only one patient died while admitted to the hospital, and six out of 160 patients died within 30 days (43). Because of this low mortality rate, the effect of TTFA on mortality could not be sufficiently analyzed. Therefore, the primary effect measure is the development of septic shock (43). Isaranuwatchai et al. (33) targeted both post-chemotherapy cancer patients as well as general populations presenting with fever or symptoms suggesting of infection. Regarding patients with confirmed febrile neutropenia, no significant association was found between mortality and antimicrobial therapy delivered before or after 1 h (33). However, the study did find a significant association when treating patients with cancer in general, but without neutropenia. Mortality was rare also in this study, with four out of 149 patients (33). The combination of few and small studies, as well as only a portion of the patients having a clinical sepsis diagnosis, makes it impossible to draw a proper conclusion regarding the effect of timely antibiotic treatment on patients with febrile neutropenia.

To summarize, prompt treatment is essential when treating septic patients. Based on the findings in this systematic review, the recommendations proposed in *The Surviving Sepsis Campaign* appear to be reasonable goals (4). However, it is still essential to keep in mind that focus on prompt antimicrobial therapy could lead to misdiagnosis of infection focus and is related to a worse outcome (27). Another possible consequence is unnecessary exposure to broad-spectrum antibiotics. Although we have found supporting evidence for *the Surviving Sepsis Campaign* in this study, findings in the different systematic reviews are still conflicting. A systematic review and meta-analysis by Huang et al. (7), supports the need for prompt treatment, expressing that each hour of delay in antibiotic administration was associated with increased odds of mortality in adult patients with sepsis. Another systematic review and meta-analysis by Sterling et al. (54) studied the potential mortality benefit regarding patients with severe sepsis or septic shock, receiving antimicrobials within 3 h of triage or 1 h of shock recognition. Nevertheless, no significant mortality benefit was found.

### Implications of the results for practice, policy, and future research

Based on our findings, we support the Surviving Sepsis Campaign’s recommendations regarding antibiotic administration within one hour for patients with septic shock or high likelihood of sepsis and 3 h for patients with suspected sepsis without shock (4). As the results show a significant difference in mortality linked to an hourly delay in TTFA, prompt administration of antibiotics saves lives. Thus, screening for infectious agents and collection of blood cultures should be prioritized before administering treatment (4). The 3 h window for patients with suspected sepsis without shock allows time to consider differential diagnoses, identify possible sources of infection, and collect necessary samples. However, prompt administration of antibiotics should remain a priority. For further practice and research, focusing on how diagnostic processes and routines can be made more efficient, allowing for more rapid initiation of antibiotic treatment, is also essential.

In the treatment of neutropenic patients at risk of developing sepsis, current guidelines recommend administering antibiotics within 2 h of fever onset (55). Thus, only five articles on the topic were included in the review; the majority of our studies on neutropenic sepsis demonstrated a significant increase in mortality related to time to antibiotic administration – two specifically with each hour of delay (43, 44), and one showing a significant difference before and after the 2 h mark (25). Like sepsis in the general population, these findings underscore the critical importance of prompt antibiotic treatment. Given the vulnerability of immunocompromised patients, timely antibiotic administration is likely even more crucial in this group. However, interpreting results and drawing conclusions regarding neutropenic sepsis with such a limited sample size and mortality rate, should be done with caution. As previously mentioned, the majority of patients included in the neutropenic studies did not have clinical sepsis, and none of studies in our search investigated only neutropenic sepsis exclusively. It is important to address patients with neutropenic sepsis as a clinically distinct population, and whom current guidelines are often extrapolated, despite limited direct evidence. This highlights the need for more research to strengthen the guidelines further and explore potential improvements, ensuring the best possible outcomes for these patients.

### Strengths and limitations

In working with our review, we found no other reviews that categorize sepsis studies based on the three sepsis definitions and neutropenic sepsis, possibly making our review the first of its kind. This review has several other strengths, including following the PRISMA 2020 checklist, making our review more transparent and complete while minimizing bias. The checklist sets high standards for methodological reporting, improving this review’s accuracy and overall quality. Our eligibility criteria and search strategy included studies reporting sepsis, time to first antibiotics and outcomes, clearly addressing which studies to exclude. To reduce bias, we double-blinded our selection process, data extraction, and risk of bias assessment, reducing observer bias and improving validity and objectivity. We also reread the studies several times to capture important details. We cross-checked other systematic reviews and the Surviving Sepsis Campaign Guidelines to ensure no relevant studies were missed during the literature search. Finally, we organized tables displaying the various time cut-offs for outcomes across different studies, making it easier for authors and readers to conclude.

The review also has several limitations. Firstly, we conducted a literature search using a single database, PubMed. A search across multiple databases would have been beneficial to ensure a broader foundation for our findings. Secondly, different types of bias might be present in this systematic review. Most of the studies were assessed as high quality according to the Newcastle Ottawa Scale, indicating a relatively low risk of bias. However, the Newcastle Ottawa scale has its limitations. The assessment of bias relies heavily on subjective considerations, with somewhat vague criteria. Different authors will therefore potentially come to different conclusions regarding the same studies. Neither does the scale cover all relevant types of bias, for example reporting bias. Further, we chose not to assess reporting bias and the certainty of evidence due to the nature of our narrative synthesis. The heterogeneity in populations, definitions and outcomes across studies made a structured assessment challenging. This leaves room for undetected reporting bias, potentially leading to conclusions made on misguided information. In this systematic review, we have classified the studies by sepsis definitions. Studies with unclear sepsis definitions have been classified based on inclusion criteria, which may have introduced misclassification bias. However, the rationale behind the classification has been discussed in the “Materials and methods” section. Furthermore, publication bias must be considered. Studies with positive results have a higher likelihood of getting published and could lead to an overestimation of effect. Thirdly, we chose to conduct a narrative systematic review rather than a meta-analysis due to the heterogenous nature of sepsis and the varied outcomes in the included studies. A meta-analysis might have provided greater statistical power and would potentially have made our conclusion even more accurate.

Furthermore, the literature search primarily yielded observational studies and included only one RCT matching our inclusion criteria. The observational studies generally lack clear time cutoffs for administrating antibiotic treatment. Conducting RCTs could provide more precise insights into the timing of antibiotic administration and observe outcomes in relation to timing. On the other hand, this would be considered unethical, as it would involve withholding necessary antibiotics from patients at risk of severe outcomes. Additionally, one could argue that the heterogeneity within the septic population – regarding factors such as comorbidities, age, and sex – might complicate the evaluation of RCT results. Hence, the absence of RCTs might limit our ability to draw definite conclusions about the time to first administer antibiotics and patient outcomes.

An interesting point would be to present a detailed breakdown of crude mortality rates across time intervals (< 1, 1–3, 3–6, > 6 h) for all studies. An overview of how crude mortality varies with time to antibiotic treatment could present information regarding trends in mortality in sepsis patients, as well as identifying and comparing differences in the included studies. However, such data are inconsistently reported across the included studies. Presenting them uniformly could therefore be misleading or incomplete.

Lastly, we noted that most of the studies in our analysis did not distinguish between sepsis, severe sepsis, and septic shock and instead calculated odds ratios for the entire population. The lack of differentiation made it more challenging to directly compare our findings with the one-hour and three-hour targets outlined in the Surviving Sepsis Campaign guidelines (4).

## Conclusion

In this article, we aimed to investigate the timing of antimicrobial therapy initiation and its consequences. We have reviewed the existing literature regarding the timing of antimicrobial administration and its impact on septic patients to evaluate whether the Surviving Sepsis Campaign’s 1 h target for initiating antibiotics, is a reasonable goal (4).

Based on the findings in this systematic review, the recommendations proposed in *The Surviving Sepsis Campaign* appear to be beneficial in patients with septic shock and a suspected sepsis diagnosis (4). In patients where the diagnosis of sepsis is uncertain, the guidelines recommend investigating other options, while antibiotics are administered within 3 h (4). Allowing time to consider other causes for disease could help prevent misdiagnosis and unnecessary exposure to broad-spectrum antimicrobials. Our conclusion is based on a systematic review with a narrative synthesis instead of a meta-analysis, due to the heterogeneity of sepsis. Most studies found a significant association between mortality and delay in antibiotic administration with a 1 h mark or hourly cutoffs. This indicates strongly that antimicrobials received before 1 h reduce mortality, though several of the included studies did not come to the same conclusion. The heterogeneity in sepsis manifestation makes it difficult to provide guidelines appropriate for all patients and could explain why studies have such varying results. In the treatment of neutropenic patients at risk of developing sepsis, current guidelines recommend administering antibiotics within 2 h of fever onset (55). Due to few studies on the subject, concluding is impossible. Though given the vulnerability of immune-compromised patients, it is likely that timely antibiotics administration is even more crucial in this group. More research is needed to further strengthen the guidelines, and to explore potential improvements.

## Data Availability

The original contributions presented in this study are included in this article/Supplementary material, further inquiries can be directed to the corresponding author.
