# Supplementary material for: Timing of antibiotic initiation in sepsis and neutropenic fever
Source: Front Med (Lausanne). 2025 Sep 2;12:1597047. doi: 10.3389/fmed.2025.1597047 (PMC12436305; doi:10.3389/fmed.2025.1597047)
Supplement: Supplementary file 1 [file Data_Sheet_1.pdf]

Supplementary Material

1 Supplementary Data

Supplementary Material should be uploaded separately on submission. Please include any supplementary data, figures and/or tables.

Supplementary material is not typeset so please ensure that all information is clearly presented, the appropriate caption is included in the file and not in the manuscript, and that the style conforms to the rest of the article.

2 Supplementary Figures and Tables

For more information on Supplementary Material and for details on the different file types accepted, please see here.

2.1 Supplementary Figures

Supplementary File 1. Search strategy

Search element	Sepsis	Antibiotics	Time-to-treatment
MeSH	"Sepsis"[Mesh:NoExp] OR "Bacteremia"[Mesh] OR "Shock, Septic"[Mesh]	"Anti-Bacterial Agents"[Mesh]	"Time-to-Treatment"[Mesh]
Free text (title, abstract)	Sepsis OR Septic* OR Endotoxin Shock* OR Bloodstream Infection* OR Blood Poisoning OR bacteremia*	Antibiotic* OR anti-bacterial agent*	Time-to-Treatment* OR time to treatment* OR Door-to-Treatment OR door to treatment* OR delay* treatment OR treatment delay*

Supplementary File 2. Flowchart illustrating excluded articles. Additionally, one duplicate and one inaccessible study were excluded.

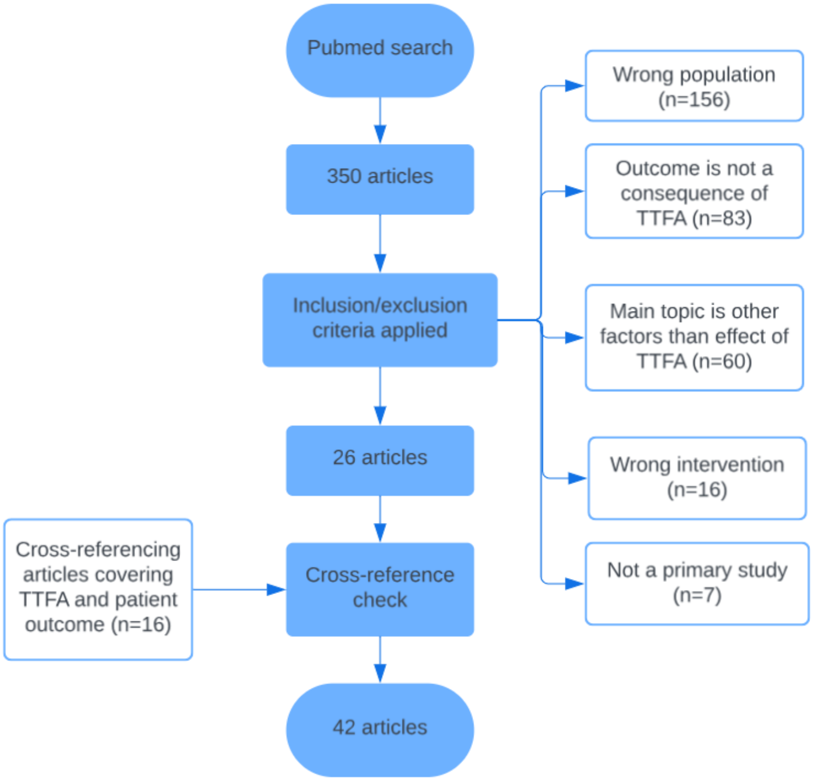

Supplementary File 3 Quality appraisal of included studies using the Newcastle-Ottawa scale

	Selection				***Comparability***		***Outcome***			***Total score***	***Newcastle-Ottawa***
Bisarya R, et al. (28)	*	*	*	*	*	*	*	*	*	9/9	High
Yang A, et al. (29)	*	*	*	*	*	*	*	*	*	9/9	High
Seymour C, et al. (30)	*	*	*	*	*	*	*	*	*	9/9	High
Liu VX, et al. (31)	*	*	*	*	*	*	*	*	*	9/9	High
Ferrer R, et al. (11)	*	*	*	*	*	*	*	*	*	9/9	High
Kumar A, et al. (23)	*	*	*	*	*	*	*	*	*	9/9	High
Ko BS, et al. (39)	*	*	*	*	*	*	*	*	*	9/9	High
Abe T, et al. (24)	*	*	*	*	*	*	*	*	*	9/9	High
Rhee C, et al. (13)	*	*	*	*	*	*	*	*	*	9/9	High
Joo YM, et al. (40)	*	*	*	*	*	*	*	*	*	9/9	High
Corl KA, et al. (32)	-	*	*	*	*	*	*	*	*	8/9	High
Suberviola CB, et al. (33)	*	*	*	*	*	*	*	*	*	9/9	High
Puscarich MA, et al. (17)	*	*	*	*	-	*	*	*	*	8/9	High
Gaieski DF, et al. (18)	*	*	*	*	*	*	*	*	*	9/9	High
Garnacho-Montero J, et al. (34)	-	-	*	*	*	*	*	*	*	7/9	High
Wisdom A, et al. (27)	*	*	*	*	-	*	*	*	*	8/9	High
Andersson M, et al. (44)	*	*	*	*	*	*	*	*	*	9/9	High
Han X, et al. (35)	*	*	*	*	*	*	*	*	*	9/9	High
Taylor SP, et al. (42)	*	*	*	*	*	*	*	*	*	9/9	High
Peltan ID, et al. (19)	*	*	*	*	*	*	*	*	*	9/9	High
Pruinelli L, et al. (12)	*	*	*	*	*	*	*	*	*	9/9	High
Rüddel H, et al. (15)	*	*	*	*	-	*	*	*	*	8/9	High
Bloos F, et al. (RCT) (10)	*	*	*	*	-	*	*	*	*	8/9	High
Whiles BB, et al. (9)	*	*	*	*	*	*	*	*	*	9/9	High
Im Y, et al. (20)	*	*	*	*	*	*	*	*	*	9/9	High
Schinkel, et al. (43)	*	*	*	*	*	*	*	*	*	9/9	High
Seymour CW, et al. (36)	*	*	*	*	*	*	*	*	*	9/9	High
Sivayoham N, et al. (14)	*	*	*	*	-	*	*	*	*	8/9	High
Siewers K, et al. (41)	*	*	*	*	*	*	*	*	*	9/9	High
Seok H, et al. (26)	*	*	*	*	*	*	*	*	*	9/9	High

Ogawa K, et al. (21)	-	*	*	*	-	*	*	*	*	7/9	High
Daniels LM, et al. (22)	*	*	*	*	*	*	*	*	*	9/9	High
Sung B, et al. (25)	*	*	*	*	-	*	*	*	*	8/9	High
Goldman JD, et al. (37)	*	*	*	*	-	*	*	*	*	8/9	High
Morneau K, et al. (38)	*	*	*	*	*	*	*	*	*	9/9	High
Isaranuwatchai S, et al.(33)	*	*	*	*	*	*	*	*	*	9/9	High
Philippon A, et al. (45)	*	*	-	*	-	*	-	*	*	6/9	Moderate
Liang CY, et al. (32)	-	*	*	*	*	*	*	*	*	8/9	High
Londoño J, et al. (48)	*	*	*	*	*	*	*	*	*	9/9	High
Peltan ID, et al. (46)	*	*	*	*	*	*	*	*	*	9/9	High
Tantarattanapong S, et al. (18)	-	*	*	*	-	-	*	*	*	6/9	Moderate
Bulle EB, et al. (31)	*	*	*	*	*	*	*	*	*	9/9	High
